# Novel Function of lncRNA ADAMTS9-AS2 in Promoting Temozolomide Resistance in Glioblastoma via Upregulating the FUS/MDM2 Ubiquitination Axis

**DOI:** 10.3389/fcell.2019.00217

**Published:** 2019-10-02

**Authors:** Yuanliang Yan, Zhijie Xu, Xi Chen, Xiang Wang, Shuangshuang Zeng, Zijin Zhao, Long Qian, Zhi Li, Jie Wei, Lei Huo, Xuejun Li, Zhicheng Gong, Lunquan Sun

**Affiliations:** ^1^Department of Pharmacy, Xiangya Hospital, Central South University, Changsha, China; ^2^National Clinical Research Center for Geriatric Disorders, Xiangya Hospital, Central South University, Changsha, China; ^3^Department of Pathology, Xiangya Hospital, Central South University, Changsha, China; ^4^Department of Neurosurgery, Xiangya Hospital, Central South University, Changsha, China; ^5^Key Laboratory for Molecular Radiation Oncology of Hunan Province, Center for Molecular Medicine, Xiangya Hospital, Central South University, Changsha, China

**Keywords:** glioblastoma, TMZ resistance, ADAMTS9-AS2, FUS, MDM2, ubiquitination

## Abstract

**Background:**

LncRNAs have been shown to play essential roles in cancer therapeutic response. However, the detailed mechanism of lncRNAs in temozolomide (TMZ) resistance in glioblastoma (GBM) remain to be elucidated.

**Methods:**

To elucidate the mechanism maintaining TMZ resistance, we constructed two TMZ-resistant GBM cell lines (T98G-R/U118-R). LncRNAs from four public datasets were reanalyzed, and the candidate lncRNA ADAMTS9-AS2 was evaluated in TMZ-treated GBM patients and *in vitro* cell lines.

**Results:**

Reanalysis of lncRNA expression profiles identified ADAMTS9-AS2 as significantly overexpressed in TMZ-resistant GBM cells and as positively associated with the IC_50_ of TMZ in GBM cells. Overexpression of ADAMTS9-AS2 was also significantly associated with poor TMZ response and shorter progression-free survival (PFS) in TMZ-treated GBM patients. Knockdown of ADAMTS9-AS2 inhibited proliferation and attenuated the IC_50_ of TMZ, as well as mitigating invasion and migration in TMZ-resistant GBM cells. Subsequent investigations indicated that reduced expression of ADAMTS9-AS2 significantly suppressed expression of the FUS protein, which was predicted as a direct substrate of ADAMTS9-AS2. Expression trends of FUS were directly correlated with those of ADAMTS9-AS2, as shown by increasing concentrations and prolonged treatment with TMZ. RNA pull-down and RIP assays indicated that both endogenous and exogenous ADAMTS9-AS2 directly binds to the RRM and Znf_RanBP2 domains of FUS, consequently increasing FUS protein expression. Knockdown of ADAMTS9-AS2 reduced the half-life of FUS and decreased FUS protein stability via K48 ubiquitin degradation. Moreover, the E3 ubiquitin-protein ligase MDM2 interacts with and down regulates FUS, while the RRM and Znf_RanBP2 domains of FUS facilitate its binding with MDM2. ADAMTS9-AS2 decreased the interaction between MDM2 and FUS, which mediates FUS K48 ubiquitination. Additionally, knockdown of the ADAMTS9-AS2/FUS signaling axis significantly alleviated progression and metastasis in TMZ-resistant cells.

**Conclusion:**

ADAMTS9-AS2 possessed a novel function that promotes TMZ resistance via upregulating the FUS/MDM2 axis in GBM cells. The RRM or Znf_RanBP2 domains of FUS facilitate the combination of ADAMTS9-AS2 and FUS, competitively inhibiting MDM2-dependent FUS K48 ubiquitination and resulting in enhanced FUS stability and TMZ resistance. Our results suggest that the ADAMTS9-AS2/FUS/MDM2 axis may represent a suitable prognostic biomarker and a potential target in TMZ-resistant GBM therapy.

## Introduction

Glioblastoma (GBM), the most lethal form of primary intrinsic brain tumor in both pediatric and adult populations, has the highest mortality rate among all malignant nervous system neoplasms, with a median survival of only 12–15 months. In 2005, the US Food and Drug Administration (FDA) approved combination temozolomide (TMZ) and radiotherapy treatment in adults with newly diagnosed glioblastoma, as well as the use of TMZ alone as a maintenance treatment (Hombach-Klonisch et al., 2018). TMZ remains the most widely used and effective first-line chemotherapeutic for GBM patients, with high bioavailability and tolerability. However, over time, the majority of patients with GBM gradually develop resistance to TMZ during treatment, leading to GBM recurrence and treatment failure (Kaur et al., 2018; Teng et al., 2018).

Several pathways have been elucidated that regulate TMZ resistance, and expression of the DNA repair protein O^6^-methylguanine-DNA-methyltransferase (MGMT) is considered the predominant cause of TMZ resistance. MGMT expression can be silenced by methylation of the promoter/enhancer region, and polymorphisms in MGMT gene promote MGMT expression and TMZ resistance (Wang et al., 2017; Chen X. et al., 2018). Other plausible mechanisms are involved in TMZ resistance as well, including activation of base excision repair (BER), reduced activity of mismatch repair (MMR) genes, histone posttranslational modifications, GBM stem cells and dysregulation of other effectors (Garnier et al., 2018). Epigenetic variations have been shown to play major roles in mediating the resistance to targeted therapies and conventional cytotoxic agents. Aberrant miRNA expression, such as in mIR-93, noted in clinically relevant tumor subtypes of GBM, influences tumor response to therapy through epigenetic miRNA-based silencing or sensitizing effects (Huang et al., 2019). Recent studies suggest that long non-coding RNAs (lncRNAs) are indispensable for the regulation of cellular processes in glioma tumorigenesis and in therapeutic responses (Yan et al., 2017). Specifically, several clinically relevant lncRNAs have been correlated with patient outcome in GBM and mediate biological functions, including stemness, immunity, development, regulation of gene expression, and regulation of protein synthesis (Schmitt and Chang, 2016). Thus, understanding the most relevant mechanisms of TMZ resistance may help identify novel drug targets and more effective therapies.

The discovery of lncRNAs has provided insight into the underlying biological mechanisms of glioma phenotypes, which is mediated through their interactions with other cellular macromolecules, including proteins, RNA and DNA (Peng et al., 2018). LncRNA nuclear-enriched abundant transcript 1 (NEAT1) contributes to glioma cell growth and invasion through the WNT/β-catenin pathway by scaffolding the EZH2 protein (Chen Q. et al., 2018). Findings from Sa’s group have shown that lncRNA homeobox transcript antisense intergenic RNA (HOTAIR) promotes drug delivery across the blood tumor barrier (BTB) in glioma treatment by sponging miR-148b-3p (Sa et al., 2017). Chen reported that lncRNA AC003092.1 may act as an endogenous “sponge” of miR-195, promoting expression of TFPI-2 and overcoming TMZ resistance in glioma cells (Xu et al., 2018). Through chromatin modification, LncPRESS1 disrupts deacetylation of H3K56 by sequestering SIRT6 from chromatin to safeguard pluripotency-specific stem cells (Jain et al., 2016). Using RNA expression profiling, Mazor G et al. found that lncRNA TP73-AS1 comprises a clinically relevant lncRNA that influences metabolism-related genes and ALDH1A1, conferring TMZ resistance to GBM stem cells (Mazor et al., 2019). In addition, studies have shown that lncRNA metastasis-associated lung adenocarcinoma transcript 1 (MALAT1) plays a promising role in TMZ therapeutic response to GBM (Amodio et al., 2018). Nanocomplex-mediated silencing of MALAT1 effectively sensitizes glioma cells to temozolomide therapy (Kim et al., 2018). Furthermore, ML Zhang et al. showed that circRNAs generated from lncRNA LINC-PINT, containing short open reading frames, encode functional peptides that suppress oncogenic transcriptional elongation in glioblastoma (Zhang et al., 2018). However, it remains largely unknown how specific lncRNAs influence in the mechanical properties of glioma cells in response to TMZ exposure.

To address these challenges, we reanalyzed lncRNA profiles in TMZ-resistant glioma cells using four public glioma-associated lncRNAs datasets. Our data identified 12 differentially expressed lncRNAs in TMZ-resistant glioma cells. Among these, lncRNA ADAMTS9-AS2 (ADAM metallopeptidase with thrombospondin type 1 motif 9 antisense RNA 2) was significantly overexpressed. Moreover, alterations in ADAMTS9-AS2 were correlated with TMZ response in glioma patients. Using subsequent functional assays, ADAMTS9-AS2 was found to be involved in fused in sarcoma (FUS)/MDM2 mediated progression in TMZ-resistant GBM.

## Materials and Methods

### Cell Culture

T98G and U118 human glioma cell lines, identified by the short tandem repeat (STR) analysis (Supplementary Data Sheet S1), were obtained from the Cancer Research Institute, Central South University, China, and have been authenticated by short tandem repeat genotyping (Genesky Biotechnologies Inc., Shanghai). As described in our previous study (Dai et al., 2017), we established T98G-R and U118-R TMZ-resistant cell lines with continuous stepwise selection using increasing concentrations of TMZ for greater than 6 months. Next, the half maximal inhibitory concentration (IC_50_) was determined to confirm stable resistance to TMZ. Glioma cells and HEK 293T cells were maintained in DMEM supplemented (C11995500, HyClone) with 10% fetal bovine serum (10099141C, Gibco) and 1% penicillin/streptomycin (10378016, Gibco) at 37°C with 5% CO_2_.

### Reagents

TMZ was purchased from Sigma-Aldrich Corporation Chemicals (PHR1437). Cycloheximide (CHX) was purchased from MedChemExpress (HY-12320). The proteasome inhibitor MG132 was purchased from Selleck Chemicals (S2619). All reagents were dissolved in dimethylsulfoxide (Amresco). Specific siRNAs and smart silencer (smsiRNAs) RNAs were purchased from Ribo (China) with sequences shown in Supplementary Table S1. Full-length ADAMTS9-AS2 and MDM2 were PCR-amplified from human cDNA and subcloned into pcDNA3.1(+) to create ADAMTS9-AS2 expression plasmids. A FUS expression plasmid, along with truncation constructs used in this study, were purchased from Addgene (29609, 29610, 29611, 29612) and Vigene Biosciences. The constructs His-Ubiquitin (WT) and His-Ubiquitin (K48R) were kind gifts from Prof. Gang Huang (Cincinnati Children’s Hospital, Cincinnati, OH, United States). Overexpressing plasmids (1 μg) or smsiRNAs/siRNAs (100 nM) of indicated genes were transfected into cells using Lipofectamine 3000 (L3000015, Invitrogen) for overexpression and knockdown of indicated genes, respectively, followed by analysis 48–72 h later.

### Human Tissues

One hundred forty-four glioma tissues were collected between 2015 and 2018 from the Department of Neurosurgery, Xiangya Hospital of Central South University. This project was approved by the Chinese Clinical Trial Register (ChiCTR-RPC-16008569) and the ethics committee of Xiangya Hospital (Changsha, China). Data do not contain any information that could identify patients. Detailed clinical information was collected from patient records and is listed in Supplementary Table S2. Samples were obtained from patients during surgery and were immediately snap-frozen in liquid nitrogen until use. Chemotherapy response status was assigned to patients based on progression-free survival (PFS) in GBM patients with TMZ treatment (Stupp et al., 2015, 2017) denoted as TMZ response (no recurrence within 4 months after surgical resection) and TMZ non-response (recurrence within 4 months after surgical resection).

### MTS Assays and IC_50_

T98G, U118, and their TMZ-resistant cell lines were seeded in 96-well plates (2 × 10^3^ cells/well) and cultured overnight. Various concentrations of TMZ (0, 0.2, 2, 20, 200, 2,000 μM) or/and indicated transfection reagents were added into the medium for 72 h. Then, MTS assays were performed to determine cell viability following the manufacturer’s protocols. Briefly, 20 μl MTS solution (G358C, Promega) was added into each well and incubated for 6 h. Absorbance was detected at 490 nm using a VICTOR X2 microplate reader (PerkinElmer, United States). IC_50_ was determined using sigmoidal concentration-response curve-fitting mode using SPSS software. Variable slope was employed to calculate IC_50_ values [non-linear regression; dose-response-inhibition; log(inhibitor) vs. response- variable slope (4 parameters)]. Cell proliferation was determined by treating TMZ-sensitive or TMZ-resistant glioma cells (1 × 10^3^ cells/well) with 100 μM TMZ for 5 days in 96-well plates. Cell proliferation of TMZ-sensitive or TMZ-resistant glioma cells (1 × 10^3^ cells/well) after indicated transfection was determined for 5 days in 96-well plates.

### Cell Cycle Analysis

After serum starvation and cell cycle synchronization for 12 h, cells were seeded into T25 flasks at 1 × 10^6^ cells each. As cells adhered to the plate, drug treatments were added to the flask, and cells were incubated for 48 h. For each condition, detached and adherent cells were harvested, fixed in 70% ethanol at −20°C for at least 12 h, and incubated with propidium iodide (20 μg/ml), phosphate-buffered saline (PBS) and RNase A (50 μg/ml) in the dark. Stained cells were detected using flow cytometry (Guava easyCyte 8HT, Millipore, United States).

### Scratch Assay

The scratch assay was used to measure cell migration *in vitro*. In brief, cells were seeded into 60 mm dishes (2.5 × 10^5^ cells/well) and cultured until they formed a fused monolayer for 24 h. The smsiRNAs or/and siRNAs treatments were administered for each group according to the experimental design. A P200 pipette tip was used to create a scratch. After 48 h, wound closure was imaged using a microscope with mounted camera. Relative migration distance was measured, using ImageJ software, by determining the fraction of cell coverage across the scratch. Relative migration distance was calculated as follows: (%) = migration area/total area × 100%.

### Transwell Assay

The transwell assay was used to measure cell invasion *in vitro*. Matrigel (BD Biosciences, NJ, United States) was mixed with medium in a ratio of 1:8 and placed on the upper surface of each insert in 24-well transwell plates (BD Biosciences, NJ, United States). The chambers were held 6 h in the incubator. In brief, 1 × 10^4^ cells were added to the upper chamber, and 10% FBS was added to the lower chamber. The smsiRNAs or/and siRNAs treatments were administered for each group according to the experimental design. After 48 h, chambers were fixed and stained with 0.05% crystal violet for 2 h. Cells on the upper surface were gently scraped, and stained cells were imaged and quantified under the microscope. The ImageJ software was used to assist cell counting in cell invasion assays.

### RNA Extraction and qPCR

Total RNA was extracted using TRIzol reagent (Invitrogen) according to the manufacturer’s instructions. The RNA quality was checked after 1% agarose gel electrophoresis with ChemiDoc XRS system (Bio-Rad, United States) and using Protein nucleic acid spectrophotometer (Beckman Coulter, United States). The A260/A280 ratios of RNA are allowed between 1.8 and 2.2. Total RNA was then reverse-transcribed to cDNA using the PrimeScript^TM^ strand cDNA synthesis kit (6210, Takara). The PARIS Kit (AM1921, Invitrogen) was used to separate nuclear and cytoplasmic RNAs in GBM cells. The qPCR reaction was then performed by CFX96 Touch Real-Time PCR Detection System (Bio-Rad, United States) to determine the expression levels of targets, and performed in triplicate in three independent experiments. The primer sequences of qPCR are shown in Supplementary Table S1. Changes in target mRNA levels relative to a reference gene (β-actin) were determined using the 2^–ΔΔ*ct*^ method with iTaq Universal SYBR green Supermix as previous reported (1725121, Bio-Rad) (Song et al., 2019).

### RNA Pull-Down Assay

LncRNA ADAMTS9-AS2 was transcribed *in vitro* from the pcDNA3.1(+) vector using the T7 RiboMAX large-scale RNA production system (P1300) and was biotin labeled using the Pierce RNA 3′ end biotinylation kit (20160). Two milligrams of protein extract from T98G-R cells were then mixed with 100 pmol biotinylated RNA, incubated with nucleic-acid-compatible streptavidin magnetic beads and washed (Pierce magnetic RNA-Protein pull-down kit, 20164). Proteins that bound to the streptavidin-coupled dynabeads were resolved using reducing sample buffer and then subjected to western blot.

### RNA Immunoprecipitation (RIP)

RIP analysis was performed using the Magna RIP RNA-Binding Protein Immunoprecipitation Kit (Millipore, Bedford, MA, United States) according to the manufacturer’s instructions. Antibodies to FUS and the V5-tag used for RIP were the same as those used for western blot. C-immunoprecipitated RNAs were detected by strand specific qPCR.

### Western Blot Analysis

Whole cell protein was isolated using Pierce IP Lysis Buffer (Thermo Fisher Scientific, United States). The PARIS Kit (AM1921, Invitrogen) was used to separate nuclear and cytoplasmic protein fractions in GBM cells. Protein concentrations were quantified using the Micro BCA Protein Assay Kit (23229, Thermo Scientific). Purified proteins were boiled with 4 × loading buffer, and denatured protein samples were separated by SDS-PAGE on 10% polyacrylamide gels. Then, samples were transferred to NC membranes (HATF00010, Millipore). After blocking with 5% non-fat milk for 1 h, membranes were probed with appropriate primary antibodies overnight at 4°C. The next day, membranes were washed with PBS/Tween (PBST) and incubated with appropriate secondary antibodies for approximately 1 h at room temperature. Protein bands were visualized by Immobilon Western chemiluminescent reagents (WBKLS0500, Millipore). Information for utilized primary antibodies is shown in Supplementary Table S3.

### Immunoprecipitation (IP)

For ubiquitination assays, cells were transfected according to the experimental requirements followed by treatment with 1 μM MG132 for 6 h. Then, 500 μg protein lysates were incubated with 1 mg/ml specific primary antibody with gentle rocking for 3 h at 4°C. Protein A/G beads (10002D, Invitrogen) were subsequently added to precipitate protein complexes and further incubated with gentle rocking overnight at 4°C. Precipitates were collected, and supernatants were discarded. Pellets were fixed and resuspended in SDS sample loading buffer before boiling. Samples were then subjected to SDS-PAGE for western blot analysis.

### Data Acquisition and Reanalysis Using Different Bioinformatics Methods

The bioinformatics analysis of lncRNA profiling in GBM tissues was conducted through several independent bioinformatics databases. LncRNA Modulator Atlas in Pan-cancer (LncMAP) is a user-friendly web platform, providing lncRNA-mediated transcriptional signatures in human cancer tissues (Li et al., 2018). Using the Cancer RNA-Seq Nexus (CRN), we selected two studies (CRN Glioma and CRN Glioblastoma) to explore the lncRNA profiles between the normal and GBM tissues (Li J. R. et al., 2016). The Atlas of Non-coding RNAs in Cancer (TANRIC) was used for the further confirmation of the changes of lncRNAs in GBM (Li et al., 2015). Then, the consistently dysregulated genes were identified using a Venn analysis and Heatmap diagram.

In addition, to evaluate the roles of lncRNAs in protein complexes, we used the RAID database to version 2.0 (RAID v2.0) (Yi et al., 2017), which integrated diverse RNA-associated interactions. Using this tool, we explore the proteins that bind to disregulated lncRNAs. Furthermore, another integrated bioinformatics platform, UbiBrowser (Li et al., 2017), was used to investigate the human E3 ubiquitin-protein ligase-substrate interaction network.

### Statistical Analysis

Statistics were calculated using SPSS 20.0 (SPSS Inc., United States). One-way ANOVA (Kruskal–Wallis test) and Chi-square tests were employed to analyze differences in demographic characteristics and clinical data among different groups. Receiver operating characteristic (ROC) curves were established to discriminate GBM response patients from non-response patients. Area under the ROC curve was used as an accuracy index for evaluating the predictive performance of the hypothesized lncRNA. ^∗^*p* < 0.05 and ^∗∗^*p* < 0.01 were regarded as statistically significant.

## Results

### Establishment of T98G-R and U118-R TMZ-Resistant GBM Cell Lines

**FIGURE 1:**
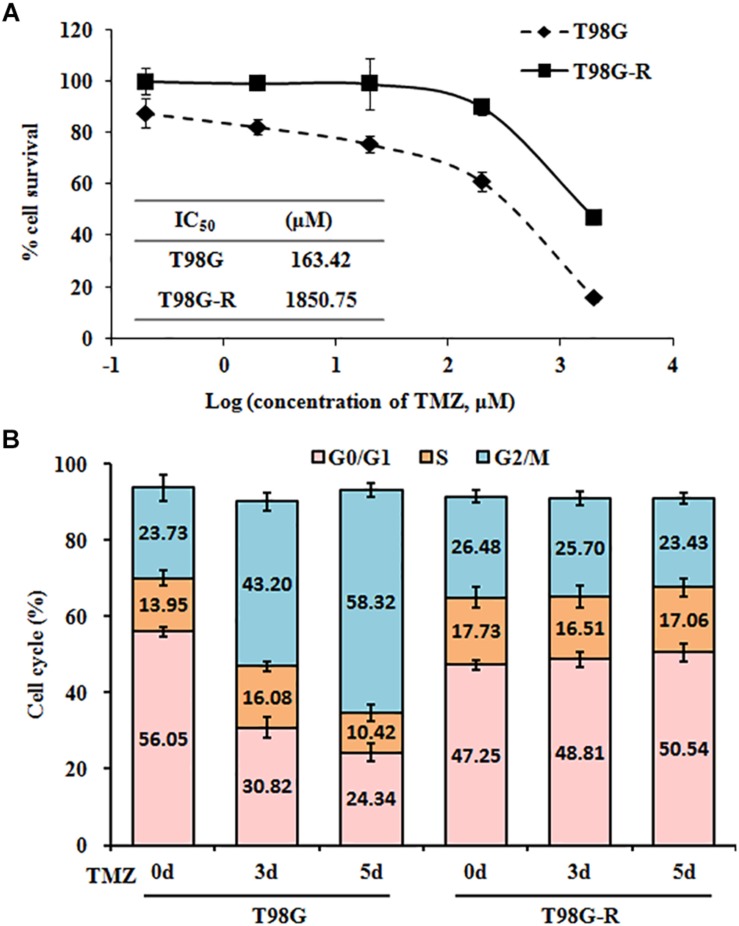
Establishment and characterization of TMZ-resistant GBM cell lines. **(A)** Cell viability and IC_50_ analysis was performed to evaluate cytotoxicity of TMZ to T98G and T98G-R cells in response to the indicated concentrations of TMZ for 72 h. **(B)** T98G and T98G-R cells were treated with 100 μM TMZ for 3 and 5 days, and cell cycle was examined by flow cytometry. The above experiments were repeated independently three times with similar results. Each data point represents mean ± SD.

To confirm resistant phenotypes, T98G-R and U118-R cancer cells were exposed to various doses of TMZ. After approximately 72 h, we measured cell viability and IC_50_ using MTS assay. Compared to the parental cells, T98G and U118, cell viability rates in T98G-R and U118-R cells were much higher in response to TMZ treatment (Figure 1A and Supplementary Figure S1A). IC_50_ values of TMZ-resistant cells were increased more than fourfold compared to parental cells (T98G vs. T98G-R: 163.4 μM vs. 1850.7 μM; U118 vs. U118-R: 87.6 μM vs. 383.6 μM) (Figure 1A and Supplementary Figure S1A). To further compare the proliferation capacity of parental and TMZ-resistant GBM cells, the cells were treated with 100 μM TMZ for 3 or 5 days. Significantly different cell cycle distribution was observed between TMZ-resistant cells and their parental cell lines. Upon TMZ treatment, T98G and U118 cells caused marked cell cycle arrest in G2/M phase in a time-dependent manner, while there were no significant changes in T98G-R and U118-R cells (Figure 1B and Supplementary Figure S1B). These findings are consistent with the TMZ-resistant phenotype of T98G-R and U118-R cell lines.

### LncRNA ADAMTS9-AS2 Is Overexpressed in TMZ-Resistant GBM Cell Lines

**FIGURE 2:**
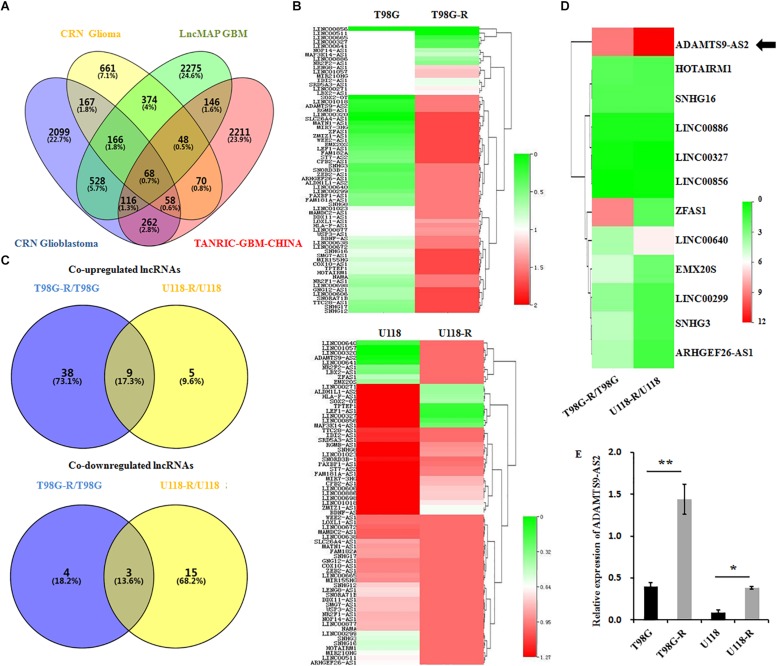
ADAMTS9-AS2 is significantly overexpressed in TMZ-resistant GBM cells. **(A)** Venn diagram of four datasets from different databases. **(B)** Changes in 68 glioma-related lncRNAs were examined between T98G/T98G-R and U118/U118-R. **(C)** The Venn diagram indicates the co-upregulated and co-downregulated lncRNAs in TMZ-resistant cells, T98G-R and U118-R. **(D)** Heatmap showing the 12 co-differentially expressed lncRNAs in TMZ-resistant cells, T98G-R and U118-R. **(E)** qPCR assay of ADAMTS9-AS2 transcript expression in TMZ-resistant GBM cell lines. Quantitative results are from three independent experiments and are shown as the mean ± SD. ^∗^*p* < 0.05, ^∗∗^*p* < 0.01.

LncRNAs are essential epigenetic regulators with critical roles in tumor initiation and malignant progression. To examine whether changes in lncRNA are involved in therapeutic response to TMZ in GBM cells, we first performed data mining using CRN Glioma (Li J. R. et al., 2016), CRN Glioblastoma (Li J. R. et al., 2016), TANRIC-GBM-CHINA (Li et al., 2015), and LncMAP GBM (Li et al., 2018) datasets and evaluated overlapping lncRNAs in GBM samples. The Venn diagram revealed that approximately 68 lncRNAs are common among these four published datasets (Figure 2A). After a preliminary screen through strand-specific qPCR, we identified 12 co-differentially expressed lncRNAs in TMZ-resistant T98G-R and U118-R cells. Among these, nine lncRNAs were frequently upregulated, and three were frequently downregulated (Figures 2B,C). Furthermore, the ADAMTS9-AS2 was the most consistently and markedly overexpressed RNA transcript in TMZ-resistant T98G-R and U118-R cells (*p* < 0.01, *p* < 0.05, respectively) (Figures 2D,E). Thus, in subsequent experiments, we primarily evaluated the functional roles of ADAMTS9-AS2 in TMZ response in GBM samples.

### Overexpressed ADAMTS9-AS2 Predicts Poor TMZ Response in GBM Patients

**FIGURE 3:**
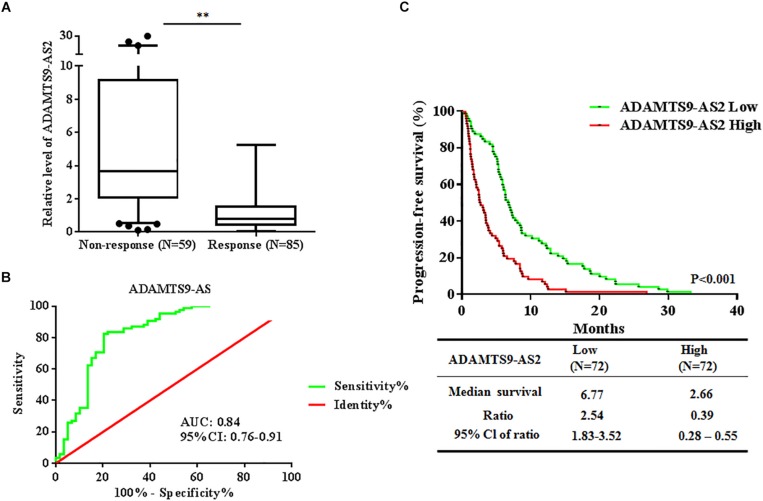
Altered expression of ADAMTS9-AS2 is associated with TMZ response in 144 GBM patients. **(A)** qPCR assay of ADAMTS9-AS2 transcript expression in patients with differential TMZ response status. **(B)** ROC curves were established to discriminate TMZ responding patients from non-responding patients. **(C)** PFS among patients with ADAMTS9-AS2 low (green, median PFS: 6.77 mouths) and ADAMTS9-AS2 high (red, median PFS: 2.66 mouths) groups. ^∗∗^*p* < 0.01 were regarded as statistically significant.

To investigate whether ADAMTS9-AS2 expression is associated with TMZ response in GBM patients, we assessed ADAMTS9-AS2 transcriptional levels in TMZ response and TMZ non-response GBM tissues. The results revealed that ADAMTS9-AS2 was significantly upregulated in non-responding tissues compared to responding tissues (7.13 ± 1.07 vs. 1.21 ± 0.12, *p* < 0.001) (Figure 3A). A ROC curve was drawn to investigate the potential diagnostic value of ADAMTS9-AS2 expression in differentiating response status in GBM patients. The AUC was 0.84, with the diagnostic sensitivity and specificity reaching 63.87 and 69.99%, respectively (Figure 3B). Moreover, we observed improved PFS among patients with lower ADAMTS9-AS2 expression compared to those with higher ADAMTS9-AS2 expression (6.77 vs. 2.66 months, *p* < 0.001) (Figure 3C). Collectively, these clinical data on our patients support the conclusion that ADAMTS9-AS2 represents a significant prognostic marker in GBM patients after TMZ treatment.

### ADAMTS9-AS2 Induces TMZ Resistance by Regulating Metastasis

**FIGURE 4:**
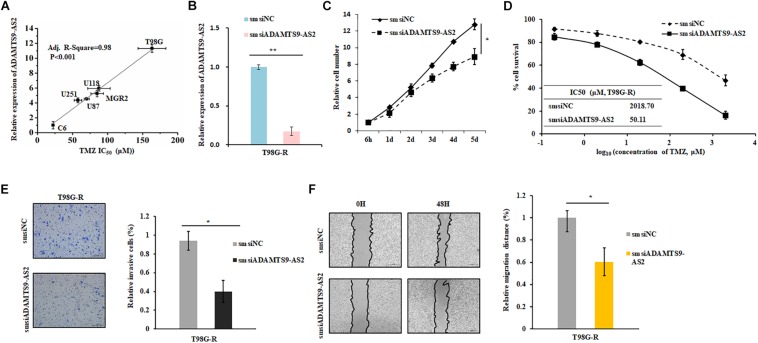
ADAMTS9-AS2 supports TMZ resistance in T98G-R GBM cells. **(A)** The correlation between ADAMTS9-AS2 mRNA expression and IC_50_ values in six GBM cells was quantified by Spearman’s rank correlation. **(B)** qPCR confirmed that smsiRNAs mediate knockdown of ADAMTS9-AS2. Relative cell number **(C)**, TMZ IC50 value **(D)**, invasion ability **(E)** and migration ability **(F)** were examined in T98G-R cells after knockdown of ADAMTS9-AS2. Quantitative results of three independent experiments are shown as the mean ± SD. ^∗^*p* < 0.05, ^∗∗^*p* < 0.01.

To investigate the effects of ADAMTS9-AS2 on TMZ-resistant behaviors, we determined the Spearman correlation between ADAMTS9-AS2 levels and TMZ sensitivity (IC_50_) in 6 GBM cell lines (T87G, U118, MGR2, U251, U87, C6). IC_50_ values of TMZ were positively correlated with ADAMTS9-AS2 expression levels in these glioma cells (Spearman *r* = 0.98, *p* < 0.001) (Figure 4A). Next, we used a smsiRNAs-mediated knockdown strategy to inhibit ADAMTS9-AS2 expression in TMZ-resistant cells, T98G-R and U118-R (Figure 4B and Supplementary Figure S2A). Upon knockdown of ADAMTS9-AS2, T98G-R cells showed enhanced sensitivity to TMZ, which manifested as reduced cell proliferation rates (Figure 4C) and about an 40-fold decrease in IC_50_ (Figure 4D). Similar results were observed in U87-R cells (Supplementary Figures S2B,C). In addition, given one of the well-documented mechanisms in TMZ response involves elevated methylation of O6MeG DNA methyltransferase (MGMT) (Yan et al., 2016), we further want to evaluate whether the ADAMTS9-AS2 modulated TMZ-resistant behaviors was dependent on the MGMT methylation status in different GBM cells. Interestingly, ectopic expression of ADAMTS9-AS2 significantly upregulated the IC50 values of TMZ in both MGMT-positive cell lines T98G and U118 (Supplementary Figures S2D,E) and MGMT-negative cell lines U251 and U87 (Supplementary Figures S2F,G). These data collectively support that ADAMTS9-AS2 might represent a predictive marker of TMZ chemosensitivity in GBM cells in MGMT-independent mechanisms.

In addition, it has been previously demonstrated that the cancer cells with chemoresistance phenotype exhibit a mesenchymal phenotype with increased migration and invasion capacity compared with the parental cells. Given that metastasis might have promising roles in chemotherapeutic responses (Park et al., 2016; Ingham and Schwartz, 2017), we further assessed whether ADAMTS9-AS2 might influence invasion and migration. As expected, compared to the untreated group, ADAMTS9-AS2 knockdown significantly inhibited invasion in chemoresistant T98G-R and U118-R cells (Figure 4E and Supplementary Figure S2H). Scratch analysis showed reduced migratory ability in ADAMTS9-AS2 knock down T98G-R and U87-R cells (Figure 4F and Supplementary Figure S2I). Taken together, these finding illustrate that ADAMTS9-AS2 expression induces TMZ resistance via modulation of metastasis, and inhibition of ADAMTS9-AS2 resensitizes TMZ-resistant GBM cells to TMZ.

### ADAMTS9-AS2 Binds to FUS and Reduces Its K48-Linked Ubiquitination

**FIGURE 5:**
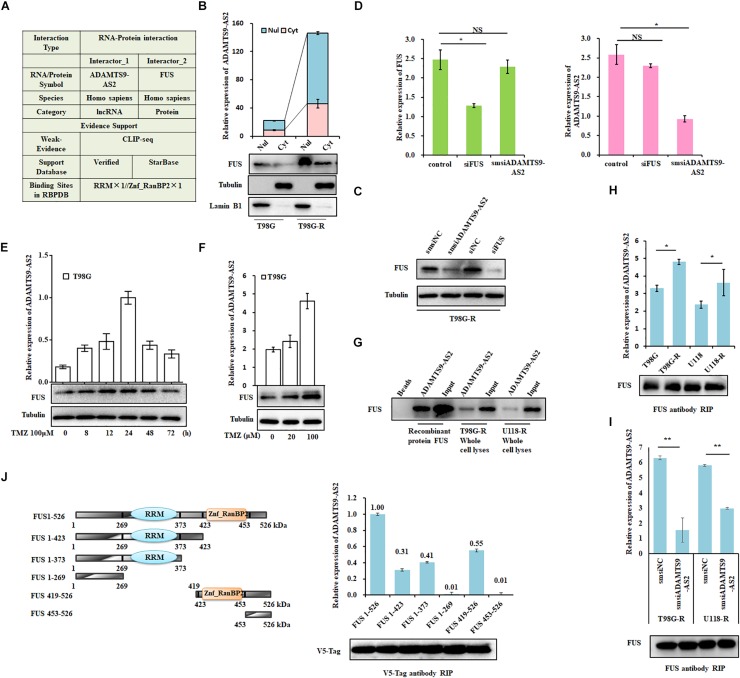
ADAMTS9-AS2 interacts with the co-localized FUS protein. **(A)** The RAID algorithm was used to predict the binding affinity of ADAMTS9-AS2 to FUS. **(B)** Subcellular localization of ADAMTS9-AS2 and FUS analyzed from nuclear and cytoplasmic extracts. **(C)** Protein levels of FUS determined by western blot analyses of lysates from ADAMTS9-AS2 knockdown cells T98G-R. **(D)** qPCR assay of ADAMTS9-AS2 and FUS transcript expression in ADAMTS9-AS2 or FUS knock down T98G-R cells. Upon different durations **(E)** or doses **(F)** of TMZ treatment, the variation tendency of ADAMTS9-AS2 and FUS was analyzed in T98G cells. **(G)** Proteins isolated from the RNA pull-down assays with biotinylated ADAMTS9-AS2 RNA were identified by western blot analyses using specific anti-FUS antibodies. mRNA isolated from the RIP assays with anti-FUS antibody was identified by qPCR using specific ADAMTS9-AS2 primers in T98G-R and U118-R cells without **(H)** or with **(I)** ADAMTS9-AS2 knockdown. **(J)** Truncated versions of V5-FUS were produced according to the predicted ADAMTS9-AS2/FUS binding domain. mRNA isolated from the RIP assays with anti-V5 tag antibody was identified by qPCR analysis using specific ADAMTS9-AS2 primers in HEK293T cells. All images displayed are representatives of three independent experiments. ^∗^*p* < 0.05, ^∗∗^*p* < 0.01.

To screen for interactions between ADAMTS9-AS2 and protein complexes that potentially act as protein scaffolds, we used the RAID algorithm (Yi et al., 2017) to identify proteins that bind to ADAMTS9-AS2. FUS, an RNA-binding protein, was identified as the main protein associated with ADAMTS9-AS2 (Figure 5A). First, we applied nuclear and cytoplasmic extraction to visualize the cellular localization and relative abundance of ADAMTS9-AS2 and FUS in TMZ-resistant cells, T98G-R and U118-R. We found that ADAMTS9-AS2 and FUS are primarily distributed in the nuclear region (Figure 5B and Supplementary Figure S3A). Furthermore, knockdown of ADAMTS9-AS2 by smsiRNAs downregulated FUS protein levels in T98G-R and U118-R cells (Figure 5C and Supplementary Figure S3B). However, no significant changes in ADAMTS9-AS2 were seen in FUS siRNA-treated cells (Figure 5D and Supplementary Figure S3C). These data indicate that FUS is a downstream effector of ADAMTS9-AS2. Resistance to TMZ is partly implicated to the low MGMT promoter methylation status and enhanced DNA repair function. To illustrated the mechanism, ADAMTS9-AS2 and FUS expression were analyzed in both MGMT-negative cell lines U251 and U87 and MGMT-positive cell lines U118 and T98G (Supplementary Figure S3D). The results showed higher expression of ADAMTS9-AS2 and FUS in relative TMZ-resistant cells than the sensitive GBM cell lines. In addition, we evaluated the effects of ADAMTS9-AS2/FUS on TMZ response in GBM cells. In response to different doses or durations of TMZ treatment, the variation of ADAMTS9-AS2 was very similar to that of FUS in the parent cells, T98G and U118 (Figures 5E,F and Supplementary Figures S3E,F), supporting the idea that the ADAMTS9-AS2/FUS axis is involved in TMZ chemotherapy.

Next, to confirm the association between ADAMTS9-AS2 and FUS, we used proteins isolated from the RNA pull-down assays and found that ADAMTS9-AS2 directly interacts with both endogenous and exogenous FUS (Figure 5G). Moreover, RIP was performed using a specific FUS antibody to ensure that ADAMTS9-AS2 was specifically immunoprecipitated from cell lysates. The binding capacity in TMZ-resistant cells was much stronger than in parent cells (Figure 5H), and knockdown of ADAMTS9-AS2 significantly weakened the association between ADAMTS9-AS2 and FUS (Figure 5I). In addition, based on the predicted binding sites of ADAMTS9-AS2 in the FUS protein sequence (Figure 5A), we obtained a series of vectors encoding V5-tagged FUS deletion mutants. RIP was performed using a specific V5 antibody to determine that ADAMTS9-AS2 specifically immunoprecipitates FUS through both the RRM and Znf_RanBP2 domains (Figure 5J). Thus, these data confirm that ADAMTS9-AS2 directly binds to FUS *in vitro*.

**FIGURE 6:**
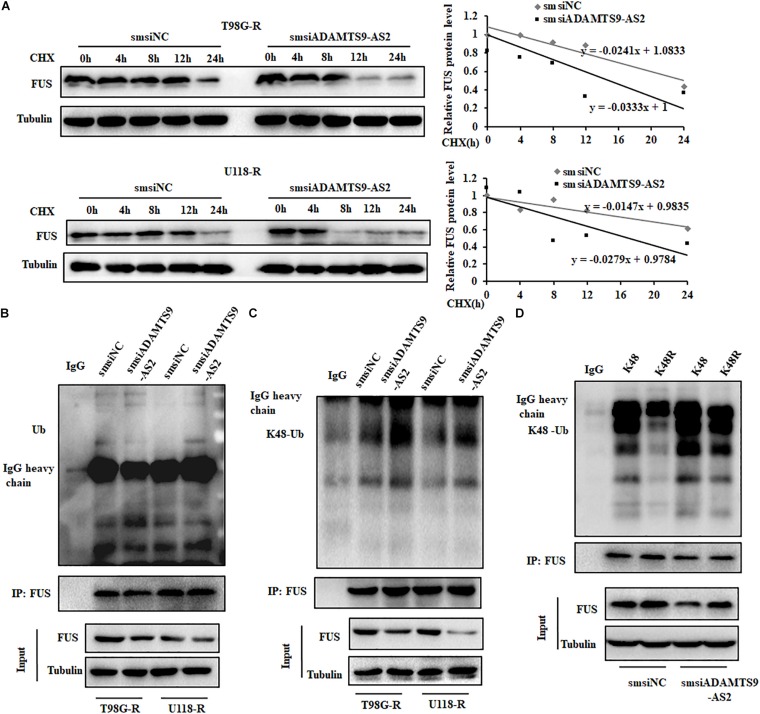
ADAMTS9-AS2 enhances FUS stability by weakening its K48-linked ubiquitination. **(A)** After treatment with CHX (20 μg/ml) for indicated times, protein levels of FUS were determined by western blot analyses of lysates from ADAMTS9-AS2 knockdown cells T98G-R and U118-R. Western blot analysis of ubiquitin **(B)** and K48-ubiquitin **(C)** immunoprecipitated with anti-FUS antibodies in T98G-R and U118-R cells. **(D)** Western blot analysis of K48-ubiquitin immunoprecipitated with anti-FUS antibodies in ADAMTS9-AS2 downregulated HEK293T cells. Experiments were repeated three times with similar results.

As FUS transcriptional levels did not change in ADAMTS9-AS2-downregulated T98G-R or U118-R cells (Figure 5D and Supplementary Figure S3C), we performed CHX chase assays to determine the protein stability of FUS. While knockdown of ADAMTS9-AS2 surprisingly attenuated the half-life of the FUS protein in TMZ-resistant cells, T98G-R and U118-R (Figure 6A), its overexpression promoted it (Supplementary Figure S4). Moreover, global and K48-linked ubiquitination of FUS in ADAMTS9-AS2-downregulated T98G-R and U118-R cells was more augmented than in controls (Figures 6B,C). In HEK293T cells transfected with ADAMTS9-AS2, V5-FUS, and K48-Ubiquitin, ADAMTS9-AS2 knockdown robustly enhanced FUS K48-linked ubiquitination, but this effect was compromised in the presence of the K48R mutant (Figure 6D). These data indicate that ADAMTS9-AS2 inhibits proteasome-dependent degradation of FUS.

**FIGURE 7:**
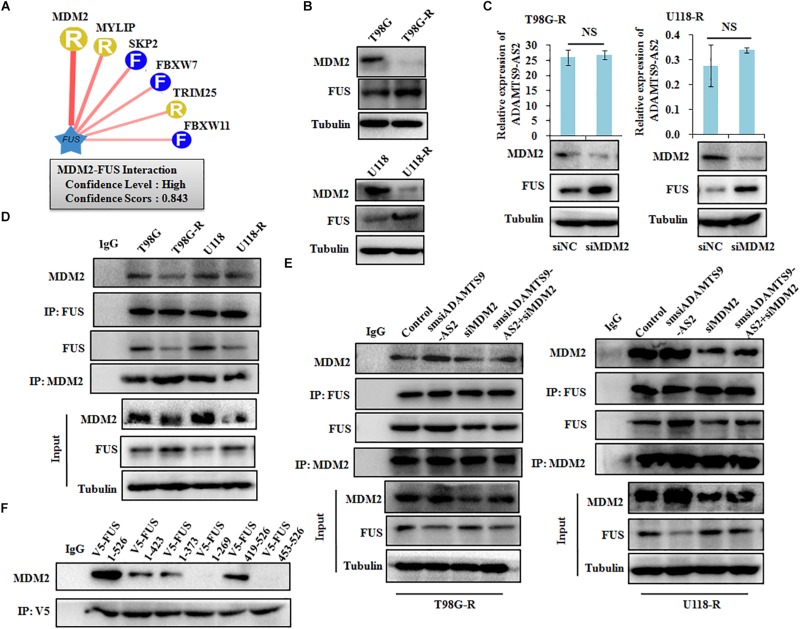
ADAMTS9-AS2 attenuates MDM2-mediated FUS ubiquitination and degradation. **(A)** The UbiBrowser tool identified the E3 ligase that interacts with FUS. **(B)** Protein levels of FUS and MDM2 were determined by western blot analyses of lysates from T98G-R and U118-R TMZ-resistant cells. **(C)** Expression levels of ADAMTS9-AS2 and FUS were analyzed in MDM2 knockdown cells T98G-R and U118-R. **(D)** IP analysis revealed the FUS/MDM2 interaction in T98G-R and U118-R cells. **(E)** IP analysis revealed the FUS/MDM2 interaction in ADAMTS9-AS2 and MDM2 downregulated cells T98G-R and U118-R. **(F)** After transfection with the truncated versions of V5-FUS in HEK293T cells, proteins isolated from IP assays using anti-V5 tag antibody were identified by western blot analyses using a specific MDM2 antibody. All data are representative of three independent experiments, and representative images are shown.

To identify characteristics of the E3 ligase that interact with FUS, we used a computational predictive system, UbiBrowser (Li et al., 2017). As shown in Figure 7A, FUS interacts most strongly with the murine double minute 2 (MDM2) E3 ligase, with a confidence score of 0.843. Western blot analysis revealed that low MDM2 levels and high FUS levels are clearly seen in T98G-R and U118-R cells (Figure 7B). Furthermore, FUS expression was upregulated in MDM2-downregulated cells, whereas there was no significant change in ADAMTS9-AS2 transcription (Figure 7C). Moreover, the FUS-MDM2 interaction was decreased in T98G-R and U118-R cells (Figure 7D). Knockdown of MDM2 weakened its FUS binding ability, leading to upregulated FUS expression (Figure 7E). We further scanned the interaction between MDM2 and FUS when ADAMTS9-AS2 was knocked down. The FUS/MDM2 interaction was enhanced in ADAMTS9-AS2 knock down T98G-R and U118-R cells (Figure 7E). Then, after full-length and truncated FUS proteins were expressed in HEK293T cells, IP analysis determined that all of FUS fragments containing the RRM or Znf_RanBP2 domains specifically immunoprecipitated MDM2 (Figure 7F). Together, these results demonstrate that ADAMTS9-AS2 might attenuate the interaction between FUS and MDM2, inhibiting MDM2-mediated FUS K48-ubiquitination and degradation.

### The Effect of Modulating ADAMTS9-AS2/FUS on TMZ Chemosensitivity

**FIGURE 8:**
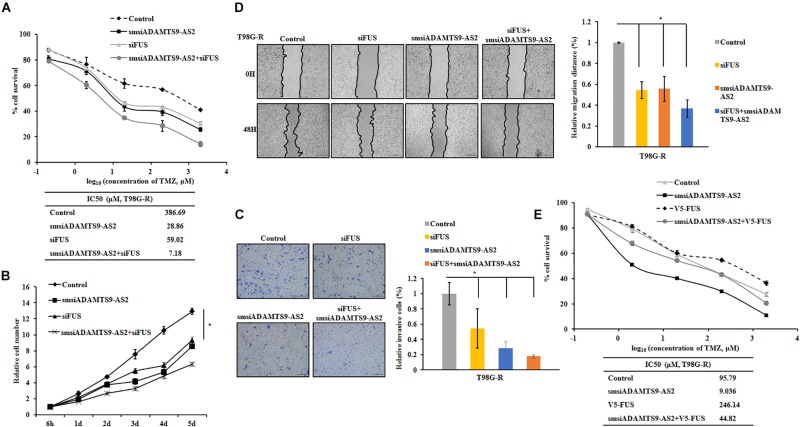
Knockdown of ADAMTS9-AS2/FUS promotes TMZ chemosensitivity in T98G-R cells. TMZ IC_50_ value **(A)**, relative cell number **(B)**, invasion **(C)**, and migration **(D)** were examined in T98G-R cells after knockdown of ADAMTS9-AS2 and FUS. **(E)** FUS overexpression could rescue the inhibitory effects of ADAMTS9-AS2 knockdown in T98G-R cells. The above experiments were repeated independently three times with similar results. ^∗^*p* < 0.05 were regarded as statistically significant.

To further determine the role of ADAMTS9-AS2/FUS in regulating therapeutic response to TMZ, we used T98G-R and U118-R cell lines stimulated with RNA interference silencing technology to downregulate the ADAMTS9-AS2/FUS signaling pathway. Under these conditions, IC_50_ values and cell proliferation rates were both significantly inhibited in ADAMTS9-AS2 or FUS downregulated cells (Figures 8A,B and Supplementary Figures S5A,B), indicating enhanced TMZ chemosensitivity. In addition, transwell and scratch assay showed similar effects for ADAMTS9-AS2 and FUS knockdown with significant inhibition of migration and invasion in both T98G-R and U118-R cells (Figures 8C,D and Supplementary Figures S5C,D). Moreover, combined knockdown of ADAMTS9-AS2 and FUS further promoted the sensitivity of TMZ-resistant cells to TMZ (Figures 8A–D and Supplementary Figures S5A–D). However, overexpression of FUS could rescue the inhibitory effects of ADAMTS9-AS2 knockdown in both T98G-R and U118-R cells (Figure 8E and Supplementary Figure S5E). These data indeed support the conclusion that ADAMTS9-AS2 inhibition enhances the antitumor effect of TMZ in GBM cells by down-regulating FUS expression.

## Discussion and Conclusion

**FIGURE 9:**
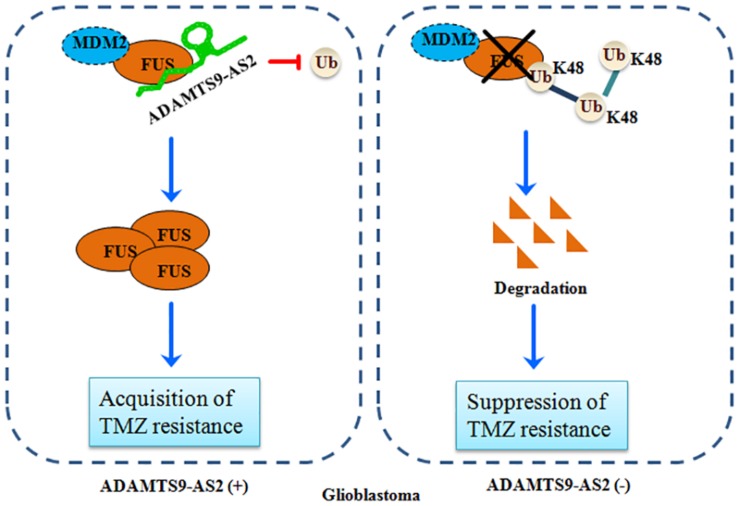
A schematic for FUS stabilization by ADAMTS9-AS2, which promotes TMZ resistance in GBM cells. ADAMTS9-AS2 binds to FUS and interferes with its MDM2-mediated K48 polyubiquitination and degradation. The FUS protein is stabilized by ADAMTS9-AS2 overexpression and promotes cell metastasis, which is required for development of TMZ resistance in GBM cells.

Over the last decade, it has been increasingly demonstrated that the majority of the mammalian genome is pervasively transcribed, resulting in the production of numerous lncRNAs (Kopp and Mendell, 2018). Accumulating evidence indicates that abnormal lncRNAs play multiple roles in maintaining tumor initiation and progression, demonstrating their crucial clinical potential as biomarkers and therapeutic targets (Slaby et al., 2017; Xu et al., 2017). However, the detailed functions of lncRNAs in GBM resistance to TMZ remains to be elucidated in detail. In this present study, we demonstrated that ADAMTS9-AS2 could directly bind to FUS and interfere with its MDM2-mediated K48 polyubiquitination. FUS stabilization by ADAMTS9-AS2 overexpression promotes the cell metastatic behavior, which is required for the TMZ resistance of GBM cells (Figure 9).

Growing evidence indicates that lncRNAs regulate the expression of target genes in glioma cells. While dependent upon the cellular and environmental context, interesting conflicting outcomes have been shown regarding lncRNAs in cancer diagnose and prognosis. For example, the lncRNA MALAT1 was categorized as a tumor-suppressive gene in glioma via ERK/MAPK-mediated growth and MMP2-mediated invasiveness, while Xiong et al. found that MALAT1 enhances glioma stem cell viability and promotes gliomagenesis through suppressing miR-129 and facilitating *SOX2* expression (Han et al., 2016; Xiong Z. et al., 2018). ADAMTS9-AS2 was first identified as a novel tumor suppressor that is regulated by DNA methyltransferase-1 (DNMT1). Knockdown of ADAMTS9-AS2 by siRNA inhibited the migration of glioma cells (Yao et al., 2014). Recent studies have revealed ADAMTS9-AS2 is a double-edged sword in the initiation and malignant progression of human cancers. Compared to adjacent normal tissue, ADAMTS9-AS2 is downregulated in colorectal cancer and predicts improved prognosis in colorectal cancer patients (Li Q. et al., 2016). In contrast, ADAMTS9-AS2 levels have been found at significantly higher levels in epithelial ovarian cancer than in normal ovaries and benign ovarian cysts shown by lncRNA microarray profiling (Wang H. et al., 2016). Upregulation of ADAMTS9-AS2 facilitates cell migration and invasion via targeting miR-143-3p/integrin α6 signaling in salivary adenoid cystic carcinoma (Xie et al., 2018). However, the roles of ADAMTS9-AS2 in TMZ-resistant GBM remain unclear. We performed the first ADAMTS9-AS2/FUS combination analysis in GBM and identified ADAMTS9-AS2 as a proto-oncogene that promotes TMZ resistance through stabilizing the FUS protein. Further clarifying the functions of ADAMTS9-AS2 might uncover the nature of GBM and provide novel targets for treatment.

The RNA-binding-protein FUS, also known as translocated in liposarcoma (TLS), is a critical regulator during the characteristic pathological features of amyotrophic lateral sclerosis (Sun et al., 2015). Recently, studies have found that FUS mRNA or protein expression is upregulated in liposarcoma (Spitzer et al., 2011), breast cancer (Ke et al., 2016), cervical cancer (Zhu et al., 2018), and FUS promotes malignant progression in non-small cell lung cancer (Xiong D. et al., 2018). Functioning as an oncoprotein, FUS has been proven to be essential for the growth of prostate cancer cells by activating androgen receptor signaling (Haile et al., 2011). Reducing FUS expression significantly abrogated lncRNA NEAT1 mediated cell survival in breast cancer cells (Ke et al., 2016). FUS also regulates the expression of 19 circRNAs by binding to introns in the splice regions, such as for circ_3279 and circ_5306. In glioma, silencing of the FUS gene inhibits the proliferation and migration of neuroblastoma cells and increases their chemical sensitivity to cisplatin by promoting expression of miRNA-141 (Wang Z. et al., 2016). In glioma-exposed endothelial cells (GECs), the FUS protein combines with circ_002136, which acts as a miR-138-5p molecular sponge, upregulating SOX13 and SPON2 and increasing angiogenesis of GECs (He et al., 2019). However, the biological significance of the FUS-mediated therapeutic response is not fully understood. Our study is the first to demonstrate that ADAMTS9-AS2 interacts with FUS in the nucleus to inhibit MDM2-medicated FUS K48-ubiquitination and degradation, which inhibits migration and proliferation in GBM TMZ-resistant cells. Identifying cellular mechanism that drive GBM to be recur and TMZ resistant is critical to improving outcomes in patients. Additional studies are needed to focus on how this resistance developed during the course of GBM progression, especially the development of TMZ resistance at different time interval. Also, the addition of primary GBM cells obtained from patients are needed to determine ADAMTS9-AS2 mediated FUS/MDM2 ubiquitination axis in future studies.

This is the first study to provide a detailed characterization of the ADAMTS9-AS2/FUS/MDM2 axis in GBM TMZ resistance. A better understanding of the potential roles of lncRNAs in GBM biology, especially the characteristics of glioma patients, is of great significance for the progression of gene-targeted therapy.

## Data Availability Statement

The raw data supporting the conclusions of this manuscript will be made available by the authors, without undue reservation, to any qualified researcher.

## Ethics Statement

The studies involving human participants were reviewed and approved by the Chinese Clinical Trial Register (ChiCTR-RPC-16008569) and the ethics committee of Xiangya Hospital (Changsha, China). The patients/participants provided their written informed consent to participate in this study.

## Author Contributions

YY, ZX, and ZG performed most of the experiments, analyzed the data, and wrote the manuscript. LQ, SZ, XC, and JW helped to perform some of the experiments. ZL, ZZ, XL, and LH provided the study material and supported administrative management. LS conceived and supervised the study, and reviewed and approved the manuscript. All authors read and approved the final manuscript.

## Conflict of Interest

The authors declare that the research was conducted in the absence of any commercial or financial relationships that could be construed as a potential conflict of interest.

## Supplementary Material

FIGURE S1 Establishment and characterization of the U118-R TMZ-resistant GBM cell line. **(A)** Cell viability and IC_50_ analysis was performed to evaluate cytotoxicity of TMZ to U118 and U118-R cells in response to treatment with indicated concentrations of TMZ for 72 h. **(B)** U118 and U118-R cells were treated with 100 μM TMZ for 3 and 5 days, and cell cycle was examined by flow cytometry. The above experiments were repeated independently three times with similar results. Each data point represents mean ± SD.

FIGURE S2 ADAMTS9-AS2 supports TMZ resistance in GBM cells U118-R. **(A)** qPCR confirmed smart silencer-mediated knockdown of ADAMTS9-AS2. Relative cell number **(B)** and IC_50_ values **(C)** were examined in U118-R cells after knockdown of ADAMTS9-AS2. **(D,E)** Ectopic expression of ADAMTS9-AS2 significantly upregulated IC50 values of TMZ in MGMT-positive cell lines T98G and U118. **(F,G)** Ectopic expression of ADAMTS9-AS2 significantly upregulated IC50 values of TMZ in MGMT-negative cell lines U251 and U87. Invasion ability **(H)** and migration ability **(I)** were examined in U118-R cells after knockdown of ADAMTS9-AS2. Quantitative results shown are of three independent experiments and represent the mean ± SD. ^∗^*p* < 0.05, ^∗∗^*p* < 0.01.

FIGURE S3 ADAMTS9-AS2 upregulates the FUS protein, which is involved in TMZ response in U118-R cells. **(A)** Subcellular localization of ADAMTS9-AS2 and FUS analyzed from nuclear and cytoplasmic extracts in U118-R cells. **(B)** Protein levels of FUS were determined by western blot analyses of lysates from U118-R ADAMTS9-AS2 downregulated cells. **(C)** qPCR assay of ADAMTS9-AS2 and FUS transcript expression in U118-R ADAMTS9-AS2 or FUS knock down cells. **(D)** ADAMTS9-AS2 and FUS Protein levels were evaluated in both MGMT-negative cell lines U251 and U87 and MGMT-positive cell lines U118 and T98G. Upon different durations **(E)** or doses **(F)** of TMZ treatment, the variation tendency of ADAMTS9-AS2 and FUS was analyzed in U118 cells.

FIGURE S4 ADAMTS-AS2 regulate the FUS protein stability in parental cells. After treatment with CHX (20 μg/ml) for indicated times, protein levels of FUS were determined by western blot analyses of lysates from ADAMTS9-AS2 over-expressed cells T98G and U118.

FIGURE S5 ADAMTS9-AS2/FUS knockdown promotes TMZ chemosensitivity in U118-R cells. TMZ IC_50_ value **(A)**, relative cell number **(B)**, invasion **(C)**, and migration **(D)** were examined in U118-R cells after knockdown of ADAMTS9-AS2 and FUS. **(E)** FUS overexpression could rescue the inhibitory effects of ADAMTS9-AS2 knockdown in U118-R cells. The above experiments were repeated independently three times with similar results. ^∗^*p* < 0.05, ^∗∗^*p* < 0.01.

TABLE S1 The sequences for the primers, siRNAs and smsiRNAs.

TABLE S2 Baseline demographic and clinical characteristics according to ADAMTS9-AS2 expression.

TABLE S3 The information of indicated primary antibodies.

DATA SHEET S1 The certificates of cell authenticity by STR analysis. The data sheet contains original STR analysis results, as well as final certification report of glioma cells.

## Abbreviations

ADAMTS9-AS2: ADAM metallopeptidase with thrombospondin type 1 motif 9 antisense RNA 2
BER: Base excision repair
DNMT1: DNA methyltransferase-1
FUS: Fused in sarcoma
GBM: Glioblastoma
IC_50_: Half maximal inhibitory concentration
lncRNAs: Long non-coding RNAs
IP: Immunoprecipitation
MALAT1: Metastasis-associated lung adenocarcinoma transcript 1
MGMT: O^6^-methylguanine-DNA-methyltransferase
MMR: Mismatch repair
PFS: Progression-free survival
RIP: RNA immunoprecipitation
smsiRNAs: Smart silencer
TMZ: Temozolomide
