# Supplementary figures and images for: Novel Function of lncRNA ADAMTS9-AS2 in Promoting Temozolomide Resistance in Glioblastoma via Upregulating the FUS/MDM2 Ubiquitination Axis
Source: Front Cell Dev Biol. 2019 Oct 2;7:217. doi: 10.3389/fcell.2019.00217 (PMC6783494; doi:10.3389/fcell.2019.00217)

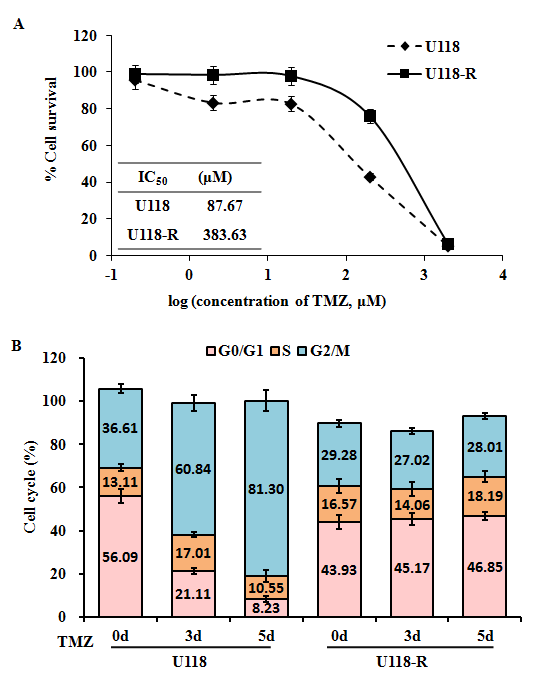

Supplement: FIGURE S1 — Establishment and characterization of the U118-R TMZ-resistant GBM cell line. (A) Cell viability and IC50 analysis was performed to evaluate cytotoxicity of TMZ to U118 and U118-R cells in response to treatment with indicated concentrations of TMZ for 72 h. (B) U118 and U118-R cells were treated with 100 μM TMZ for 3 and 5 days, and cell cycle was examined by flow cytometry. The above experiments were repeated independently three times with similar results. Each data point represents mean ± SD. [file Image_1.TIF]

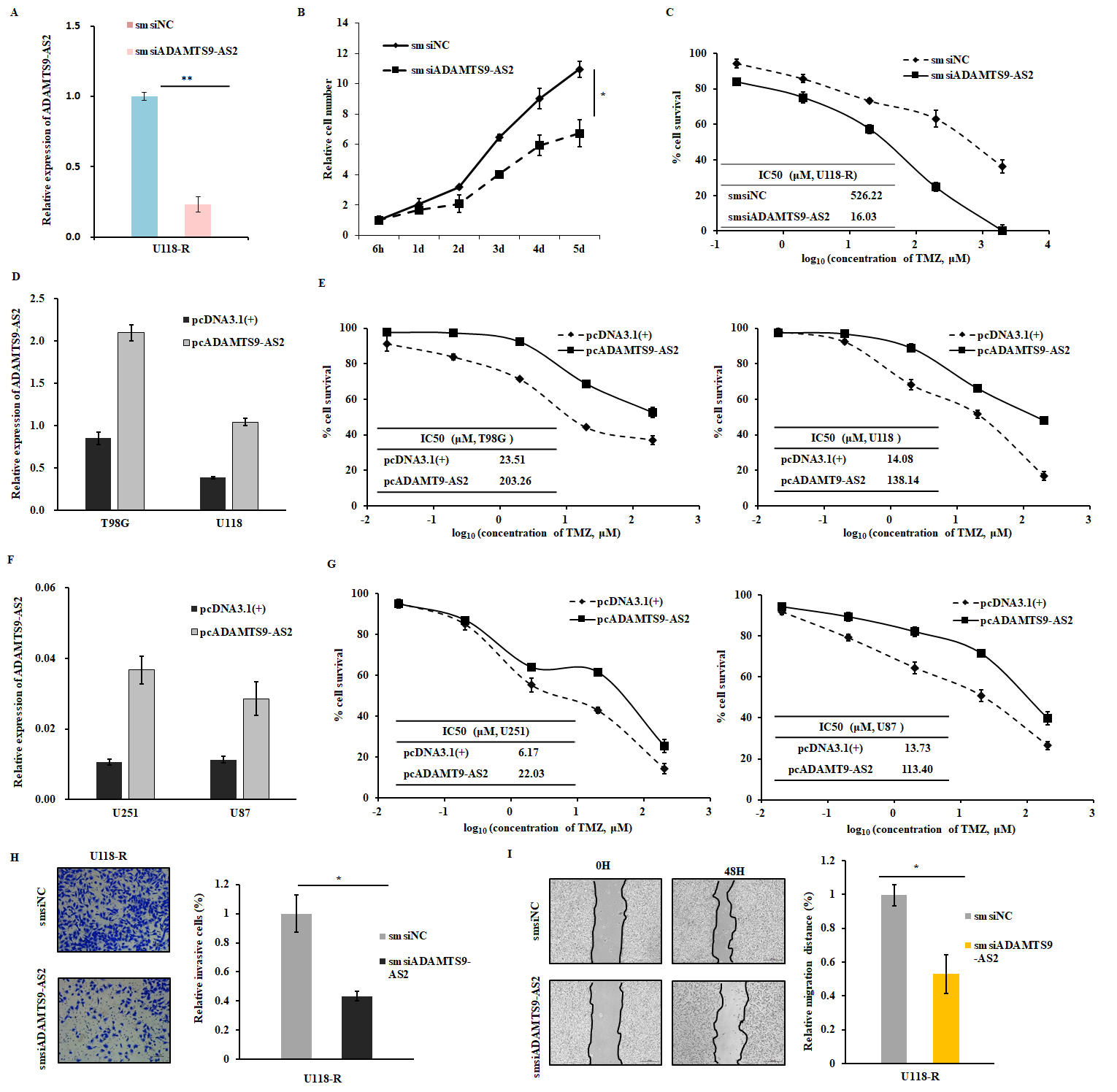

Supplement: FIGURE S2 — ADAMTS9-AS2 supports TMZ resistance in GBM cells U118-R. (A) qPCR confirmed smart silencer-mediated knockdown of ADAMTS9-AS2. Relative cell number (B) and IC50 values (C) were examined in U118-R cells after knockdown of ADAMTS9-AS2. (D,E) Ectopic expression of ADAMTS9-AS2 significantly upregulated IC50 values of TMZ in MGMT-positive cell lines T98G and U118. (F,G) Ectopic expression of ADAMTS9-AS2 significantly upregulated IC50 values of TMZ in MGMT-negative cell lines U251 and U87. Invasion ability (H) and migration ability (I) were examined in U118-R cells after knockdown of ADAMTS9-AS2. Quantitative results shown are of three independent experiments and represent the mean ± SD. ∗p < 0.05, ∗∗p < 0.01. [file Image_2.TIF]

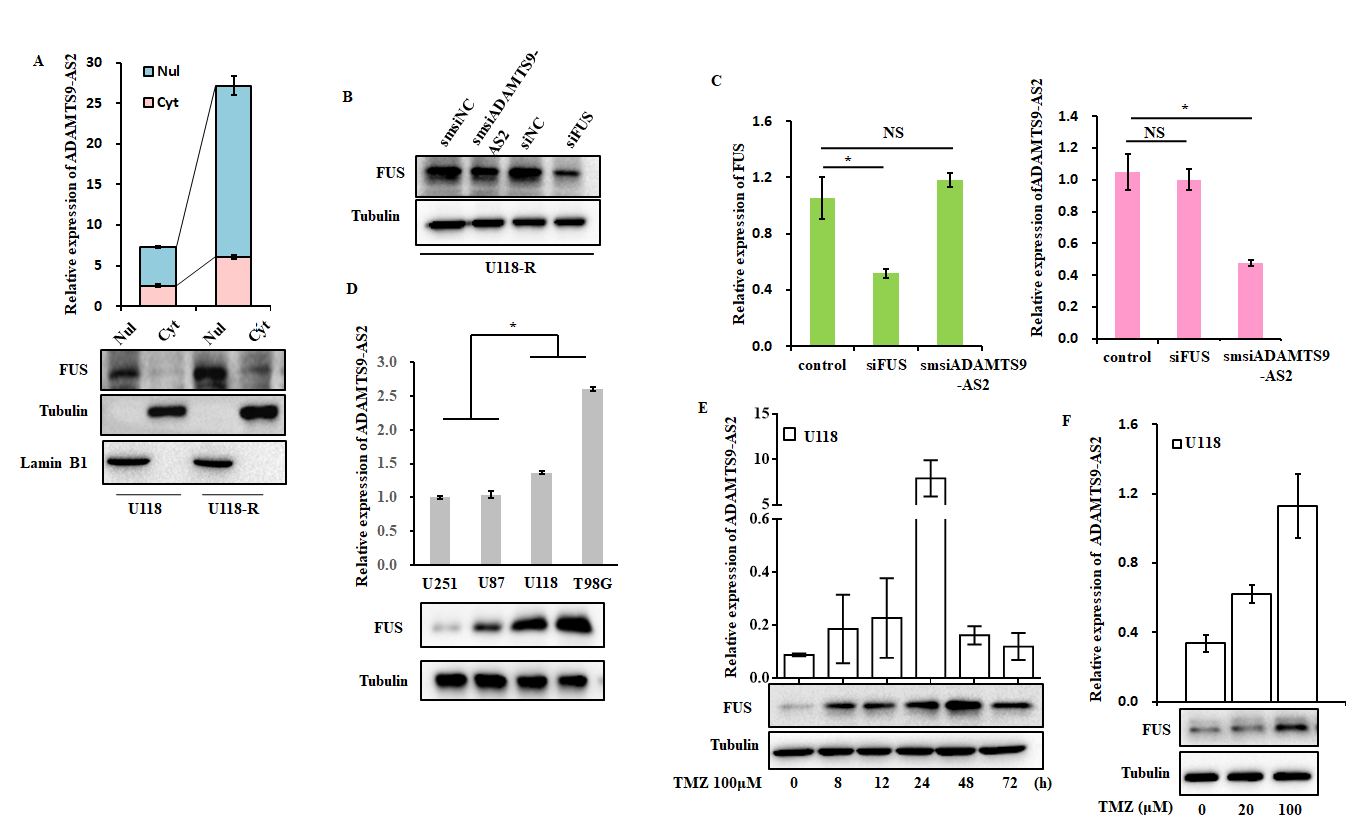

Supplement: FIGURE S3 — ADAMTS9-AS2 upregulates the FUS protein, which is involved in TMZ response in U118-R cells. (A) Subcellular localization of ADAMTS9-AS2 and FUS analyzed from nuclear and cytoplasmic extracts in U118-R cells. (B) Protein levels of FUS were determined by western blot analyses of lysates from U118-R ADAMTS9-AS2 downregulated cells. (C) qPCR assay of ADAMTS9-AS2 and FUS transcript expression in U118-R ADAMTS9-AS2 or FUS knock down cells. (D) ADAMTS9-AS2 and FUS Protein levels were evaluated in both MGMT-negative cell lines U251 and U87 and MGMT-positive cell lines U118 and T98G. Upon different durations (E) or doses (F) of TMZ treatment, the variation tendency of ADAMTS9-AS2 and FUS was analyzed in U118 cells. [file Image_3.TIF]

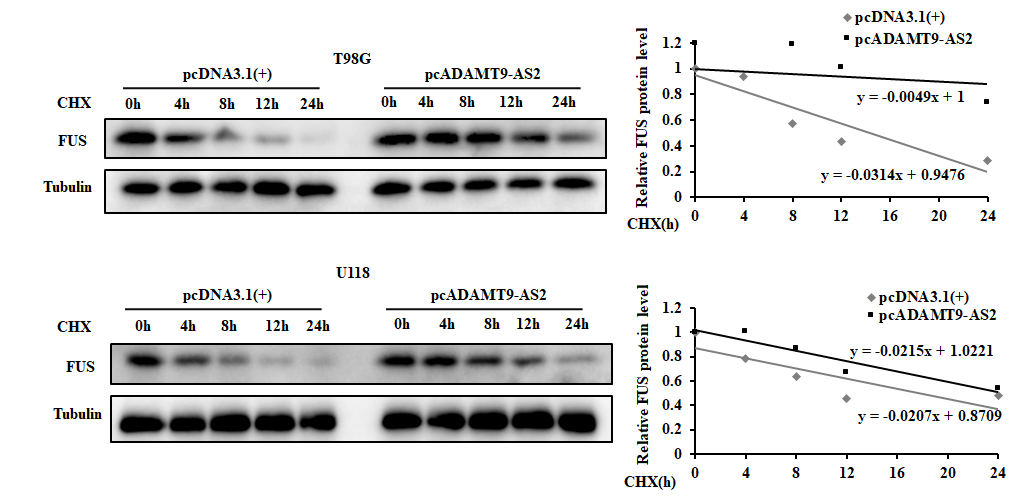

Supplement: FIGURE S4 — ADAMTS-AS2 regulate the FUS protein stability in parental cells. After treatment with CHX (20 μg/ml) for indicated times, protein levels of FUS were determined by western blot analyses of lysates from ADAMTS9-AS2 over-expressed cells T98G and U118. [file Image_4.TIF]

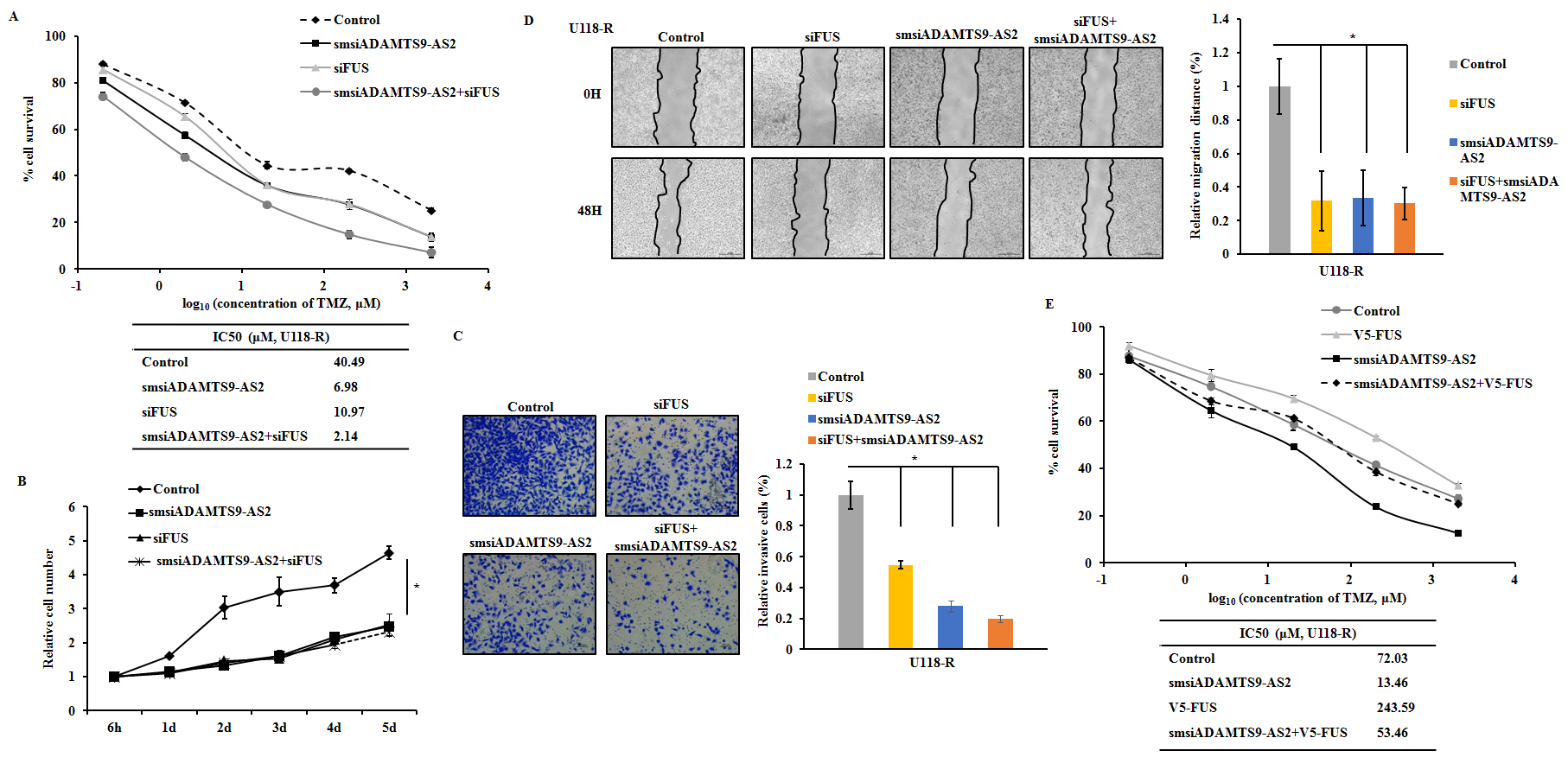

Supplement: FIGURE S5 — ADAMTS9-AS2/FUS knockdown promotes TMZ chemosensitivity in U118-R cells. TMZ IC50 value (A), relative cell number (B), invasion (C), and migration (D) were examined in U118-R cells after knockdown of ADAMTS9-AS2 and FUS. (E) FUS overexpression could rescue the inhibitory effects of ADAMTS9-AS2 knockdown in U118-R cells. The above experiments were repeated independently three times with similar results. ∗p < 0.05, ∗∗p < 0.01. [file Image_5.TIF]
