# Supplementary material for: Novel Function of lncRNA ADAMTS9-AS2 in Promoting Temozolomide Resistance in Glioblastoma via Upregulating the FUS/MDM2 Ubiquitination Axis
Source: Front Cell Dev Biol. 2019 Oct 2;7:217. doi: 10.3389/fcell.2019.00217 (PMC6783494; doi:10.3389/fcell.2019.00217)
Supplement: TABLE S1 — The sequences for the primers, siRNAs and smsiRNAs. [file Table_1.DOCX]

Table S1. The sequences for the primers, siRNAs and smsiRNAs

|  | **Primers Sequences** | |
| --- | --- | --- |
| **Symbol** | **Forward** | **Reverse** |
| TPTEP1 | TTTTTGCAGCAGCAAGATTAAGA | TCCAAATAGTCCAAATGCAAAGGA |
| FAM182A | CTGCAGCAGGAAGCCTAACT | TTGGGGTGCAGAGAAGTGTC |
| SNHG16 | CTGAGAGGCTTTGTCCTCGT | TGGTCAATTTAGGGCACGGT |
| MIR7-3HG | GAACCGCGATCTGTTTCAGC | TCCAGGGTTCTGGAGACGAA |
| ZFAS1 | TTGTCCTGCCCGTAAGTTCC | GTAGATGTCTGCACGTGGCT |
| MATN1-AS1 | GGACTGTCCAGGAGAGGAGG | CCGCTCGGAGTTATGAGCTT |
| SNHG17 | AGACATCTTCCCTGCGTTGG | GCCCAAGCTCTGAAAGCCTA |
| SNHG12 | ATGAAATGCAGGGGACCTGG | AACCTCCAGAGCTTCACACG |
| MAMDC2-AS1 | GCATTTGTGCTCCACGAGAC | TTTGGGAGAAAGCCCCCATC |
| HLA-F-AS1 | CCCCAGCTGACCTGATGAAT | ACAGTGAACACAAGTAGGCTGT |
| LINC01057 | AGGATGTGCACTTTGACGACT | TACGCCACTACACTGTTGCT |
| ZMIZ1-AS1 | CACCCACCAGTTCCCATCTC | AAAGGGAGCTAACCACACGG |
| LINC00320 | TCGTTCCCAGATTTCCTAAACTT | AAAGTTCATCTTCTTGCTGAGCC |
| ST7-AS2 | AGTTGCAGACTCCCTCTTGC | CTTGTCCAGCTTCAGGCGTA |
| LINC00606 | TCGCTAAATCTGGCCATCCC | CTCTCCTTGTCAGCGGTTGT |
| LENG8-AS1 | TTAGCTCTTTCTGCCTCCCAC | GAGTCTCAGTCAAAACAAGGCTG |
| LINC00511 | TTGGGTGAGGTACGTGAGGA | CACTGCCCTCGGAACACTAA |
| WEE2-AS1 | AAGCCCACATGAGTAGGGGA | GGCAGGCAGGTAGACATACT |
| EMX2OS | ATTGAGCAGCGGGTTAGGAT | GTTGTCCAACATTGGCCCCT |
| LINC00856 | AGTTTGCCAACTCCTGCTCT | ACCCCGTGACTTCCTTGGTA |
| LINC00271 | GGTGTCTGAGGTGGTGTAGC | GCACACAGGCACTGCTTATT |
| LEF1-AS1 | CTTCGCACTTTCTTCTGCGG | TGGTGAGTGGCCTCTAGGAA |
| GNG12-AS1 | GGCCTCCCCATGGATCATTC | TTTAAGTGCTGGGCAGGTGT |
| IDI2-AS1 | AAGACAACGCTGAAGGCTCG | CTCCCACGTCAAAGGCTGTT |
| LINC00665 | GTCCACGGGTGGGAAATTGG | TCCGGTGGACGGATGAGAAA |
| SMG7-AS1 | TGGTTTGGTCTGGTTTGGCT | GGGTGATTGGGGGCATTGTA |
| LINC00327 | CCAACACAGCATGCTTACGG | TGGATTTTCAAGGGGCTCC |
| HOTAIRM1 | TGGGCTTGGCCCAAATCTTA | GTGCACAGGTTCAAGCCATAG |
| SLC26A4-AS1 | AACGGGATCACCTGTTAGCTG | ACTCGCTTCAAGTTTGGGGA |
| MIR155HG | AGGGGTTTTTGCCTCCAACT | TCTTTGTCATCCTCCCACGG |
| SNORA71B | TTCCCCGTGTTTGAAGGGTC | CCAGGTACCAGGAGGTAACA |
| CPB2-AS1 | AGGGACCAAGCAAAACCCC | GGCAGCAAAACGTGACATCTA |
| TTC28-AS1 | GGAAAGACATGAACCAACGCC | CACACGACAGGGACGTTAAT |
| COX10-AS1 | CCGCTGTCCTGAAGAAAAAGC | CGGAGGATGCCTGTCAACT |
| LINC00299 | TTGCCAAGGACCTGCTATGC | CTCAAAACAACCCCTGCCAC |
| NR2F1-AS1 | TTGGCATGACAGTTCCCCAT | GAGGCCATTCCTCCTGCTAA |
| ZEB2-AS1 | CACACCTATTGTCTCCTGTGCT | GTGAGAAGGGGTGTCCGTG |
| PAXBP1-AS1 | AAAACATGGCCGTAGGGAGG | ATTCCACGGTTGGCAAAAGC |
| LINC00886 | ATGCGCATGAGAGTCATGGT | CCTTCTAGGGTCCCCTTTGC |
| LINC00877 | GTTGGTGAGGTGGGAGTTCA | TCTTGGGCTCCTTTTTGCCT |
| ADAMTS9-AS2 | TCTGTTGCCCATTTCCTACC | CCCTTCCATCCTGTCTACTCTA |
| SNHG3 | TGGCAGACTTGGAGCAAAAGA | TTCTGGCCAAGCAGAGTCAC |
| SOX2-OT | AGCCGAAATGGATTCACGGT | CCATGCCAGATCAGGGTGTT |
| ARHGEF26-AS1 | TTCTGCTGCAGTGGGGATTC | GACAAGAAGCCAAGCACGTC |
| LINC00698 | CAGACTGCAGCTTCCTGGTT | CCAGCAACCAGCTACGTGAT |
| BDNF-AS | CGTGTACAAGTCTGCGTCCT | AGGGAGGTCCTGGGGTATTT |
| DDX11-AS1 | TGAAATTGTTCCTGGCCCGT | AGGGAGGTGAGGTGATTCGT |
| ALDH1L1-AS2 | CAAAGAACTCCGGACCCGT | AGGCAGGAGATCCCCCATTT |
| RGMB-AS1 | CTCATCACCCGCTCGTAGAC | TTAAGGCCTTCTTGGGAGCG |
| MIR210HG | GGCAGATTTAGTGGACGCCT | TCCTACTCATCCCCAGCACA |
| NR2F2-AS1 | TGACTTGGTCACTGCAGACC | AATATCCTTCCGCCCTGCTG |
| NOP14-AS1 | ATCAAGAGGCTGCCCACATC | GCGGCTCGAATAGCCTAGAA |
| SRD5A3-AS1 | CTCCTCCCATTGGATCCGGC | CAGAGGCCATCCTTTGCTGT |
| LINC01018 | CTGAGGCCAACACTGGGAAT | GGAAAGTCCGAGACCACGTT |
| LBX2-AS1 | CGTGGGGAATGGACCCATAG | CGAGCCTTGGTCTTGTCTGT |
| LINC00641 | GCAGGGGGATGGGCTTTTA | GTGGGCTTTGTAAGGGAAACTG |
| LINC00640 | TGACTCCAATCCAGAAGACAGT | GCCTACTGTAAGTACCAATGTTCT |
| FAM181A-AS1 | AGTAGTAAGCCTCCCCACCA | GCAACCACAAGCATCCTTCA |
| LINC00638 | GTTAAGGGGTGCAGTCTCCC | AACAGCGAGGATGGTGTCTG |
| USP3-AS1 | AGCAGTCAAGCACTTTCCCA | GGCATCTTGTTTTAATGCTGCC |
| LOXL1-AS1 | TGGAGCGCGTTTAGTTCAGA | GGGCAGGTAAATGGGAACCA |
| LINC00672 | CAGATTGCACAGTCTGGGGT | CAAGTGGTTTGAGGTGGGGA |
| SNORD3B-1 | CGGCTTTTGGGTTTTCCTCG | AATAGGAGGTGCCACACAGC |
| MAP3K14-AS1 | CCACACCGCAGGTATCAGTA | TAGCCCCATGCCAGAACAAA |
| SNHG8 | AGGAAGCTGAGCTGAACACA | TAAGTCCATTGCCGGATGCT |
| NAMA | AGCAGGGCCCCATGAATAAA | TGAGGGCACTAAACAGGCAC |
| LINC01023 | CGGGGAACGGAGAAGAATCC | AAACCGAGGAAAGTGACGCA |
| LINC00202-1 | NA | NA |
| FUS | TCAATCCTCCATGAGTAGTGGT | CACGGTCCTGCTGTCCATA |
| β-Actin | CATGTACGTTGCTATCCAGGC | CTCCTTAATGTCACGCACGAT |
| ADAMTS9-AS2-T7 | GGATCCTAATACGACTCACTATAGGAAACAATCTCGACAGCGCATTT | GTAAGCCGTCACCTAGCAAAGCTTG |
|  | siRNAs Sequences | |
| FUS | ACCTTCCAATTCCTGATCACCCAAGGG | |
| MDM2 | AAGCCAUUGCUUUUGAAGUUATT | |
|  | smsiRNAs Sequences | |
| ADAMTS9-AS2 | GGTTAAAGATGTTTCAGAT  CGTGGATGTGCAATCTCTA  GGAAAGCGTCAACTATTAA  CCCACACTTGTAACCAGGCC  CCTTGTCCAATCTGCACAAA  TCTTAGTCTAAGCATGCAAC | |
