# Supplementary material for: Novel Function of lncRNA ADAMTS9-AS2 in Promoting Temozolomide Resistance in Glioblastoma via Upregulating the FUS/MDM2 Ubiquitination Axis
Source: Front Cell Dev Biol. 2019 Oct 2;7:217. doi: 10.3389/fcell.2019.00217 (PMC6783494; doi:10.3389/fcell.2019.00217)
Supplement: TABLE S2 — Baseline demographic and clinical characteristics according to ADAMTS9-AS2 expression. [file Table_2.DOCX]

| **Characteristic** | **Low expression**  **(N=72)** | **High expression**  **(N=72)** | **P-value** |
| --- | --- | --- | --- |
| Sex |  |  |  |
| Female | 30 | 27 | 0.609 |
| Male | 42 | 45 |  |
| Age (years) | 44.79±1.729 | 43.40±1.659 | 0.563 |
| Diagnosis |  |  |  |
| Newly diagnosed | 60 | 63 | 0.479 |
| Recurrence | 12 | 9 |  |
| WHO classification |  |  |  |
| III | 21 | 21 | 1.000 |
| IV | 49 | 49 |  |
| unknow | 2 | 2 |  |
| Extent of resection |  |  |  |
| Partial resection | 10 | 8 | 0.614 |
| complete resection | 62 | 64 |  |
| Antiepileptic drugs |  |  |  |
| Yes | 42 | 53 | 0.053 |
| No | 30 | 19 |  |

Table S2. Baseline Demographic and Clinical Characteristics According to ADAMTS9-AS2 expression.
