# Supplementary material for: Novel Function of lncRNA ADAMTS9-AS2 in Promoting Temozolomide Resistance in Glioblastoma via Upregulating the FUS/MDM2 Ubiquitination Axis
Source: Front Cell Dev Biol. 2019 Oct 2;7:217. doi: 10.3389/fcell.2019.00217 (PMC6783494; doi:10.3389/fcell.2019.00217)
Supplement: TABLE S3 — The information of indicated primary antibodies. [file Table_3.DOCX]

Table S3. The information of indicated primary antibodies.

| **Symbol** | **Trademark** | **Art. No** |
| --- | --- | --- |
| FUS | Santa cruz | sc-47711 |
| MGMT | Abcam | ab39253 |
| α-Tubulin | Santa cruz | sc-5286 |
| Ubiquitin | CST | #3936 |
| K48-linkage Specific Polyubiquitin | CST | #8081 |
| MDM2 | Santa cruz | sc-965 |
| Lamin B1 | CST | #12586 |
| Anti-V5 tag | Abcam | ab9116 |
