# Supplementary material for: Novel Function of lncRNA ADAMTS9-AS2 in Promoting Temozolomide Resistance in Glioblastoma via Upregulating the FUS/MDM2 Ubiquitination Axis
Source: Front Cell Dev Biol. 2019 Oct 2;7:217. doi: 10.3389/fcell.2019.00217 (PMC6783494; doi:10.3389/fcell.2019.00217)
Supplement: DATA SHEET S1 — The certificates of cell authenticity by STR analysis. The data sheet contains original STR analysis results, as well as final certification report of glioma cells. [file Data_Sheet_1.ZIP › STR genotyping/STR genotyping report.doc]

**实 验 报 告 单(Reports)**

**送样单位：湖南湘雅医院**

.

**送样细胞株(Cell lines to be analyzed)：**

n343yyL T98G

**送样人：**	**联系电话：**

**联系地址：**

**测试者(签名)：	249**

上海天昊遗传分析中心

上海天昊生物科技有限公司（盖章）

上海市浦东新区康桥路787号9号楼，邮编：201315

电话(Tel):021-50802060	传真(Fax):021-50802059	[www.geneskies.com](http://www.geneskies.com/)

Center for Genetic & Genomic Analysis, Genesky Biotechnologies. Inc.,

787 Kangqiao Road, Shanghai, 201203

**日	期(Date)：** 2018-1-04

**实验设计及操作说明(Experimental prodedures)**

利用荧光标记扩增产物长度多态分析方法对10个样本进行9个STR位点进行分型。设计了9对PCR引物，组成2个PANEL用于扩增9个多态位点。位点的荧光标记采用引物上标记5’FAM荧光标记。引物用在线Primer3 软件设计（<http://frodo.wi.mit.edu/cgi-bin/primer3/primer3_www.cgi>）。PCR产物稀释后取少量与内标标记混匀后直接上ABI3130xl进行毛细管电泳，数据文件用GeneMapper4.0（Appliedbiosystems）来分析。

**实验所用试剂和仪器：**

HotstarTaq DNA Polymerase	Qiagen

GeneScan^TM^ -500 Liz Size Standard	Applied Biosystoms

Hi-Di formamide	Applied Biosystoms

2720 Thermal Cycler	Applied Biosystoms

3130xl Genetic Analyzer	Applied Biosystoms

Centrifuge 5418D	Eppendorf

**具体实验操作步骤**

1） DNA样本取1μl 1% agarose电泳对其样本呢进行质量检查以及浓度估计，然后根据估计的浓度将样本稀释到工作浓度5-10ng/μl.

2） PCR 反应

a) PCR 引物

采用公司现有的细胞鉴定的9个STR引物体系（PANEL 1：TPOX、D16S539、TH01、CSF1PO、D5S818;PANEL 2：Amelo、vWA、D7S820、D13S317）

b) PCR 条件：

A 10µl mixture was prepared for each reaction and included 1x HotStarTaq

buffer, 3.0 mM Mg2+, 0.3 mM dNTP, 0.1µM of primer mix,1 U HotStarTaq polymerase (Qiagen Inc.) and 1 µl template DNA.

PCR程序：

The cycling program was 95ºC for 2 min;

11 cycles of 94ºC for 20 s,

62ºC-0.5 ºC per cycle for 40 s,

68ºC for 2mins;

24cycles of 94ºC for 20 s,

56 ºC for 30s,

68ºC for 2mins;

60ºC 60 min;

4 ºC for ever.

4) 取1μl PCR产物稀释80倍后，取1ul 与0.5μl Liz500 SIZE STANDARD，8.5μl Hi-Di 混匀， 95 ºC变性5分钟后上ABI3130XL测序仪

5）ABI3130xl产生的数据文件用GeneMapper4.0（Appliedbiosystems）来分析。

6）输出结果采用EXCEL进行整理、统计和分析。得出的数据结果在STR matching analysis（<http://www.dsmz.de/fp/cgi-bin/str.html>）进行数据库比对，最终得到样本的细胞株鉴定结果表

**实验结果(Results)**

1） 细胞株STR鉴定结果详见附件中的 “17C1213A Genotypes Table”文件。
2） 样本分型胶电泳图详见附件中的“截图.docx”文件。
3） 样本分型胶电泳和细胞株STR鉴定结果如下：

**分型胶电泳图**

**细胞株：n343yyL**

| **EV** | **Cell No.** | **Cell name** | **Locus names** | | | | | | | | | **Figures** |
| --- | --- | --- | --- | --- | --- | --- | --- | --- | --- | --- | --- | --- |
|  |  |  | **D5S818** | **D13S317** | **D7S820** | **D16S539** | **VWA** | **TH01** | **AM** | **TPOX** | **CSF1PO** |  |
|  | ***Query (n343yyL)*** | | ***11,11*** | ***9,9*** | ***9,9*** | ***12,13*** | ***18,18*** | ***6,6*** | ***X,Y*** | ***8,8*** | ***11,12*** |  |
| 1.00(36/36) | HTB-15 | U-118 MG | **11**,**11** | **9**,**9** | **9**,**9** | **12**,**13** | **18**,**18** | **6**,**6** | **X**,**Y** | **8**,**8** | **11**,**12** | **-** |

**细胞株： T98G**

| **EV** | **Cell No.** | **Cell name** | **Locus names** | | | | | | | | | **Figures** |
| --- | --- | --- | --- | --- | --- | --- | --- | --- | --- | --- | --- | --- |
|  |  |  | **D5S818** | **D13S317** | **D7S820** | **D16S539** | **VWA** | **TH01** | **AM** | **TPOX** | **CSF1PO** |  |
|  | ***Query (T98G)*** | | ***10,12*** | ***12,13*** | ***9,10*** | ***13,13*** | ***17,20*** | ***7,9.3*** | ***X,Y*** | ***8,8*** | ***10,12*** |  |
| 0.94(34/36) | CRL-1690 | T98G [T98-G] | **10**,**12** | **13**,13 | **9**,**10** | **13**,**13** | **17**,**20** | **7**,**9.3** | **X**,**Y** | **8**,**8** | **10**,**12** | **-** |
